# Targeted Approaches to T-Cell Lymphoma

**DOI:** 10.3390/jpm11060481

**Published:** 2021-05-27

**Authors:** Sean Harrop, Chathuri Abeyakoon, Carrie Van Der Weyden, H. Miles Prince

**Affiliations:** 1 Peter MacCallum Cancer Centre, Melbourne, VIC 3000, Australia; carrie.vanderweyden@petermac.org (C.V.D.W.); miles.prince@petermac.org (H.M.P.); 2 Monash Health, Melbourne, VIC 3168, Australia; Chathuri.abeyakoon@monashhealth.org; 3 Epworth Healthcare, Melbourne, VIC 3002, Australia; 4 Sir Peter MacCallum Department of Oncology, University of Melbourne, Melbourne, VIC 3000, Australia

**Keywords:** novel agents, monoclonal antibodies, immunotherapy

## Abstract

The T-cell lymphomas are a rare group of Non-Hodgkin’s lymphomas derived from mature T-lymphocytes. They are divided broadly into the Peripheral T-cell lymphomas and the Cutaneous T-cell lymphomas. Clinical outcomes vary widely but are generally unsatisfactory with current treatments. The development of an understanding of the various critical pathways in T-cell lymphogenesis and subsequent identification of therapeutic targets has led to a rapid expansion of the previously underwhelming T-cell lymphoma armament. This review aims to provide an up-to-date overview of the current state of targeted therapies in the T-cell lymphomas, including novel antibody-based treatments, small molecule inhibitors and immune-based therapies.

## 1. Introduction

The T-cell lymphomas are a heterogeneous group of lymphoid malignancies that can differ widely in their clinical presentation and outcome but share a predilection for poor responses to treatment with frequent early relapses and refractoriness. They are subdivided into the Peripheral T-cell lymphomas (PTCL) and the Cutaneous T-cell lymphomas (CTCL). The PTCLs are characteristically aggressive and treated with multi-agent systemic chemotherapy, like their B-cell counterparts, but often with considerably less success. Incremental benefit has been observed with the intensification of chemotherapy by the addition of further agents while the utility of stem cell transplantation remains controversial [1,2,3]. The CTCLs generally have an indolent clinical behavior with non-chemotherapy treatments favored, but ideal treatment paradigms are not well established [4].

**Figure 1:**
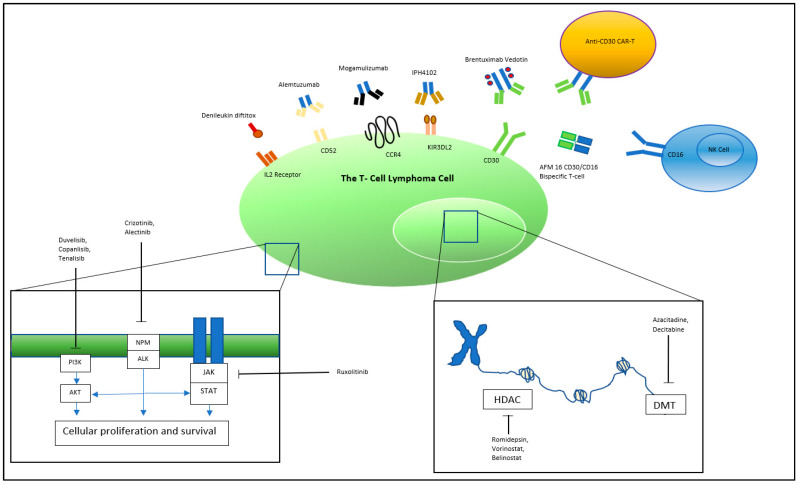
**Targets of novel T-cell lymphoma therapy.** PI3K, Phosphoinositide 3-kinase; NPM, Nucleophosmin; ALK, Anaplastic lymphoma kinase; JAK, Janus kinases; STAT, signal transducer and activator of transcription proteins; HDAC, Histone deacetylase; DMT, DNA methyltransferase.

Since the introduction of rituximab, which was approved by the FDA in 1997 for relapsed or refractory B-cell lymphoma, there has been a surge in development and approvals for novel and targeted therapies for B-cell lymphoma (Figure 1) [5]. Perhaps due to their relative rarity, there has not been the same shift in landscape in the T-cell lymphomas, despite the obvious unmet clinical need. Nonetheless, there has been considerable basic and translational research accomplished with numerous novel targeted therapies demonstrating efficacy. We review the evidence for current targeted treatments available for PTCL and CTCL.

## 2. Monoclonal Antibodies and Antibody–Drug Conjugates

### 2.1. Brentuximab Vedotin (Anti-CD30 Antibody–Drug Conjugate)

Also known as tumor necrosis factor receptor superfamily member 8 (TNFRSF8), CD30 is a transmembrane protein belonging to the tumor necrosis factor receptor (TNFR) superfamily. Expressed by activated B and T cells, it is thought to mediate signal transduction after interaction with TRAF2 and TRAF5, leading to NF-KappaB activation. With limited expression on non-malignant cells and overexpression in numerous B- and T-cell hematological malignancies, CD30 represents an ideal therapeutic target [6] (see Table 1).

Brentuximab Vedotin (BV) is an antibody–drug conjugate (ADC) combining a CD30 mAb with the microtubule inhibitor monomethylauristatin E (MMAE). After ligation of ADC with CD30 on the surface of cancer cells, MMAE binds to tubulin and disrupts the microtubule network in the cell, resulting in cell cycle arrest and apoptosis. The targeted CD30 mAb improves the stability of the microtubule inhibitor and reduces the in vivo toxicity although peripheral neuropathy is still relatively common [14].

Efficacy of BV in CD30+ PTCL was established in the phase III ECHELON-2 trial, which examined BV in combination with a chemotherapy backbone in previously untreated PTCL; this study compared the CHOP regimen (cyclophosphamide, doxorubicin, vincristine, and prednisolone) to a modified CHOP with BV incorporated in place of vincristine (A+CHP). Most patients (68%) were of the anaplastic large-cell lymphoma (ALCL) subtype, which characteristically expresses CD30. ALK+ ALCL was well represented (22%) and is associated with a better prognosis than other PTCL subtypes [6]. The addition of BV led to higher initial response rates (ORR 83% vs. 72%) with higher rates of progression-free survival (median PFS 48.2 months vs. 20.8 months) and overall survival with a 34% reduction in mortality (median OS not met). The study was not powered for comparison between different PTCL subtypes, but in an intention-to-treat population, ALK+ ALCL appeared to benefit the most with the lowest hazard ratio point estimate. Treatment discontinuations due to adverse events occurred in 6% and 7% in the A+CHP and CHOP arms, respectively [9]. The 5-year update was presented at ASH 2020, with a 5-year PFS in the A+CHP arm of 51.4% compared to 43% in the CHOP arm. Furthermore, it reported that patients that relapsed after A+CHP treatment responded to further treatment with BV even when utilized at monotherapy. In patients retreated with BV, the ORR was 59% with 38% of patients obtaining a CR [15].

The ALCL subtype has particularly benefitted from the introduction of BV, where the survival of relapsed or refractory (R/R) disease was previously poor; however, it is unclear if the incremental benefit would be seen if utilized in frontline therapy [16].

With respect to relapsed/refractory ALCL, a phase II trial has reported 5-year follow-up of 58 patients treated with single-agent BV with a 79% OS and 57% PFS at 5 years. Of the 58 patients, 38 were able to obtain a CR of which 16 proceeded to consolidative stem-cell transplant. Notably, the majority (91%) of patients that experienced peripheral neuropathy, often a dose-limiting AE, experienced resolution or improvement over time [8,17]. The phase II trial of single-agent BV reported more modest outcomes in other PTCL subtypes with an ORR of 41% and a median PFS of only 2.6 months in a cohort of 22 patients with PTCL not otherwise specified (PTCLNOS) and 13 with angioimmunoblastic T-cell lymphoma (AITL) [7].

Based on the promising phase II data demonstrating the efficacy of BV for the treatment of CD30+ CTCL [10,11], the ALCANZA phase III trial reported on the efficacy of single-agent BV compared to physician’s choice bexarotene or methotrexate in 131 patients with previously treated CTCL–mycosis fungoides (MF) and primary cutaneous anaplastic large-cell lymphoma (C-ALCL). After a median follow-up of 22.9 months, the proportion of patients achieving an objective response lasting at least four months was 56.3% compared to 12.5% in the physician’s choice arm (*p* < 0.0001). Adverse events were comparable with grade 3/4 events seen in 41% of the BV arm and 47% of the physician’s choice group [12]. A recent subanalysis demonstrated that BV remained superior to physician’s choice with no relationship between response and level of CD30 expression or presence of large-cell transformation [13]. Furthermore, a pooled analysis of five prospective trials demonstrated level of CD30 expression did not predict response to BV [18].

### 2.2. Alemtuzumab (Anti-CD52 Monoclonal Antibody)

Ubiquitous on the surface of mature lymphocytes and many other immune effector cells, such as monocytes and dendritic cells, CD52 has been shown to modulate T-cell activation via intracellular signaling pathways or by acting as an effector molecule interacting with soluble CD52 expressed on T-cell surfaces. While making CD52 an attractive target for a variety of disorders of immune cell dysregulation, such as multiple sclerosis and clonal B-cell disorders, this ubiquitous expression also results in high frequencies of toxicity, including profound cytopenia and immunosuppression-related infections [19,20,21].

Several trials have demonstrated that the addition of alemtuzumab to a CHOP backbone (CHOP-C) for the treatment of PTCL led to high rates of initial response but were hindered by significant rates of non-negligible infectious complications without significant improvement in OS [22]. The HOVON group reported particularly impressive initial response rates of around 90% with a dose intensified CHOP-C in a cohort of patients with a median age of 50 year (20–65), but without significant improvement in durability of this response compared to that expected from CHOP, while being accompanied by high rates of opportunistic infections [23]. A subsequent prospective trial of CHOP-C vs. CHOP in patients aged 61–80 reported a similar experience with excellent ORR (72 vs. 66%), but with no significant improvement in OS primarily due to treatment related toxicity [24]. Combinations with fludarabine have also been trialed with satisfactory initial responses (ORR 61%), but with no significant improvement in survival to justify the high rates of infective complication [25].

The efficacy of alemtuzumab in CTCL is perhaps even less promising with the exception of the leukemic variant, Sezary syndrome (SS). This is most likely explained by the propensity for alemtuzumab to deplete the central memory T cells that circulate between blood and skin, whereas the non-circulating effector memory T cells reside in the skin [26]. A prospective trial of alemtuzumab in CTCL in 22 patients with advanced MF/SS reported an ORR of 55% with those with erythroderma or circulating Sezary cells having a better response than those with plaque or patch disease [27]. De Masson et al. reported on a retrospective cohort of 39 patients, 23 with SS and the rest with MF, with a median follow-up of 24 months. The patients with SS had an ORR of 70% with 5 of the 23 that responded remaining progression free for greater than two years. The response rate in MF was less impressive, with only 15% of patients achieving a partial response (PR) or greater [28]. Another phase II trial in eight patients with MF/SS demonstrated modest activity (ORR 38%) in a heavily pretreated population, but with high toxicity and short PFS in responders (less than three months in all patients that achieved a PR) [29]. In an attempt to mitigate the significant infectious complications, low-dose subcutaneous alemtuzumab was utilized in a prospective trial of 14 patients with Sezary syndrome. Four patients were treated with doses up to 15 mg, with the remainder only receiving 10 mg or less. A median time to treatment failure of 12 months was reported, with twelve patients achieving a clinical response. No patient treated with 10 mg or less developed an infectious complication [30].

### 2.3. Mogamulizumab (Anti-CCR4 Monoclonal Antibody)

C-C Chemokine receptor type 4 (CCR4) is a transmembrane g protein coupled receptor that like other chemokines orchestrates cell migration. It is predominantly expressed by Th2 cells, cutaneous lymphocyte skin-homing T cells and Treg cells. CCR4 is expressed by essentially all cutaneous Th cells in human blood and skin, while one of the chemokine ligands for CCR4 (CCL17) is presented luminally by human cutaneous venules. It is likely that CCR4–CCL17 interaction contributes to skin-specific homing by triggering adhesion to endothelium [31]. Expression of CCR4 has been demonstrated in a majority of patients with Sezary syndrome, both in blood and skin, while it is present in slightly higher levels in the skin compared to normal skin in patients with mycosis fungoides [31]. CCR4 is also expressed in 30–65% of PTCLs, with expression seen in up to 90% of adult T-cell lymphoma (ATLL) [32,33].

A phase II Japanese study reported a 35% ORR in a pretreated population of 37 patients with R/R PTCL. The level of CCR4 expression did not correlate with ORR [34]. Despite this promising result, a subsequent prospective trial by Zinzani et al. of 38 patients reported a more disappointing ORR of 11.4% with poorer performance status in the latter study thought to explain at least some of the disparity [35]. Given the high level of CCR4 expression in ATLL, and the results of a promising phase II trial which reported an ORR of 50% [36], mogamulizumab was compared to investigators choice chemotherapy in a multicenter randomized trial. In a cohort of 95 patients randomized 2:1 to mogamulizumab vs. investigator’s choice, it demonstrated activity in an intention-to-treat analysis without significant toxicities (ORR 11 vs. 0%) [37]. Further combination studies are likely required before mogamulizumab can establish a role in PTCL.

The MAVORIC phase III trial compared mogamulizumab to vorinostat, an oral histone deacetylase inhibitor (HDACi), in previously treated patients with MF/SS patients. Reporting an analysis of 372 patients, they showed that mogamulizumab led to improved investigator-assessed PFS when compared to vorinostat (median PFS 7.7 vs. 3.1 months). The ORR of mogamulizumab was superior when compared to vorinostat, with the vorinostat response rates perhaps lower than expected (28.0% vs. 4.8%). Mogamulizumab also appeared to have greater efficacy in the Sezary syndrome variant than in the earlier stage CTCL groups [38]. This has led to the regulatory approval of mogamulizumab in the USA for the treatment of relapsed/refractory CTCL.

### 2.4. IPH4102 (Anti-KIR3DL2 Monoclonal Antibody)

KIR3DL2 (also known as CD158k) belongs to a family of killer-cell immunoglobulin-like receptors that can has limited expression on immune cells such as NK cell subsets and less commonly on CD8+ T cells. KIR3DL2 expression has been reported in CTCL, including Sezary syndrome, where it has been demonstrated to promote cell-death resistance [39,40]. IPH4102 is a first-in-class monoclonal antibody targeting KIR3DL2 that demonstrated a favorable safety profile and an encouraging ORR of 36.4% (43% in SS) in 44 patients with CTCL that had exposure to at least two prior systemic therapies [41]. A phase II trial (TELLOMAK) in PTCL is assessing the efficacy of IPH4102 alone and in combination with chemotherapy is currently recruiting.

### 2.5. Denileukin Diftitox (Interleukin 2—Diphtheria Toxin Recombinant Immune Toxin)

The cytokine interleukin-2 (IL2) has pleiotropic effects, as it can activate multiple different signaling pathways, including JAK/STAT, PI3K, and the MAPK/ERK. IL2 signals through the IL-2 receptor, a complex consisting of three chains, termed alpha (CD25), beta (CD122), and gamma (CD132) [42]

The IL-2 receptor is selectively expressed on activated T-lymphocytes, B-cells, and natural killer (NK) cells, and the receptor can be classified based on its affinity for IL-2. The low-affinity receptor consists of the IL-2R-α/γ (CD25, CD132) subunits that bind to IL-2 but do not cause internalization or activation. In contrast, the intermediate and high-affinity receptors that are composed of IL-2R-β/γ (CD122, CD132) and Il-2R-α/β/γ (CD25, CD122, and CD133) subunits, respectively, both cause internalization and signal transduction. It is this aspect that the fusion protein denileukin diftitox (DD) capitalizes on by replacing the receptor binding domain of the diphtheria toxin with IL2 resulting in diphtheria toxin mediated cytotoxic activity after IL2 mediated internalization [43].

Prince et al. reported the phase III trial of 144 patients with CD25 confirmed CTCL. The trial compared two dose regimens (18 and 9 mcg/kg/d) to placebo. The ORR was 43% and was dose-dependent with a higher ORR achieved in the 18 mcg/kg/d cohort (49.1%). The PFS was significantly longer (median > 2 years) for both doses when compared with the placebo [44]. A similar diphtheria toxin -IL2 Fusion Protein, E7777, is currently being investigated in phase II for safety and efficacy in R/R PTCL and CTCL. It shares the amino-acid sequence of denileukin diftitox but with a higher specific bioactivity due to greater protein purification. At last report, a total of 37 patients with a median of two prior treatments were enrolled (17 PTCL and 19 CTCL) with an ORR of 41% in PTCL and 31% in CTCL. Responses appeared independent of level of CD25 expression. Liver enzyme derangement was the most common adverse effect observed, with capillary leak syndrome seen in 11% of patients [45]

## 3. Small Molecule Inhibitors

### 3.1. ALK Inhibitors

The discovery of the fusion gene *nucleophosmin* (*NPM*)-*ALK*, a result of a t (2:5) translocation, in anaplastic large-cell lymphoma (ALCL) has led to the identification of targetable ALK fusion genes in ALCL and other malignancies, such as EML4-ALK in lung cancer [46,47]. ALK encodes a single transmembrane receptor tyrosine kinase that belongs to the insulin receptor superfamily. ALK receptor activation and function remains to be completely defined. Anaplastic lymphomas with the ALK translocation (ALK+ ALCL) are distinct entities from their ALK negative counterparts (ALK- ALCL) with unique genetic and molecular aberrancies [48]. They have a better prognosis however the survival of patients who relapsed was poor prior to the use of Brentuximab Vedotin [16].

The ALK inhibitor crizotinib was developed foremost for non-small cell lung cancer but was quickly utilized in ALK+ ALCL with impressive results [49,50]. Bossi et al. reported a phase II trial in adult patients of the first generation ALK inhibitor crizotinib in the relapsed/refractory (R/R) setting. Twelve patients with a median number of two prior lines of treatment were included with an ORR of 83.3% with 58.3% achieving a complete remission (CR). Three patients who obtained CR were resistant to previous BV administration. At the latest follow-up, seven patients (six CR and one PR) were still in response under continuous crizotinib administration, with a median duration of response of 39 months [51]. The second-generation ALK inhibitors, such as alectinib, have demonstrated superior efficacy in the ALK+ non-small-cell lung cancer setting. A Japanese phase II trial of alectinib in patients with R/R ALCL in a combined pediatric and adult population of 10 patients reported an ORR of 80%, with six complete responses. The 1-year PFS and OS were both 70% with neutropenia the most common grade 3/4 adverse effect seen in two patients [52]. Recruitment is ongoing for several promising studies, including a phase III study comparing BV to ALK inhibitors in combination with chemotherapy in the R/R setting.

### 3.2. P13K Inhibitors

Phosphatidylinositol 3-kinase (PI3K) is a lipid kinase involved in intracellular signal transduction. The PI3K-δ and PI3K-γ isoforms are preferentially expressed in leukocytes and modulate the innate and adaptive immune system. The survival and proliferative pathways of malignant lymphocytes are mediated by P13K with several studies demonstrating its role in suppressing the antitumor surveillance activity of the immune system [53,54]

Duvelisib is an oral, dual inhibitor of PI3K-δ and PI3K-γ that has demonstrated anti T-cell lymphoma activity in a phase I trial of 35 patients with T-cell lymphoma (16 PTCL and 19 CTCL) with a median number of four prior systemic treatments. The ORR for PTCL and CTCL was 50.0% and 31.6%, respectively, with three patients in the PTCL group obtaining a CR. Grade 3/4 adverse effects were common (grade 3, 79%) with the most frequent liver enzyme derangement, maculopapular rash, and neutropenia [55]. The combination of duvelisib with the histone deacetylase inhibitor (HDACi) romidepsin has also been shown to be highly active in PTCL with a manageable toxicity profile. In a multiple-arm study comparing different doses of duvelisib, the ORR across all arms was 59% in PTCL and 46% in CTCL [56].

Copanlisib is a PI3K inhibitor with a predominant activity on α and δ isoforms. A phase II trial study in R/R indolent and aggressive lymphoma included 17 patients with PTCL, of which 14 were eligible for response assessment. The ORR was 21.4%, with two patients obtaining a CR [57]. Copanlisib was subsequently combined with gemcitabine in the COSMOS phase I/II trial, which improved the response rate with an ORR and CR of 72% and 32%, respectively, and a PFS of 6.9 months. The CR rate was driven by impressive responses in the AITL subgroup with a CR rate of 55.6% [58].

The results for the Phase 1/1b trial of the highly specific, dual PI3K δ/γ inhibitor tenalisib were also encouraging. A total of 58 patients with T-cell lymphoma (28 PTCL and 30 CTCL) were enrolled with a median number of four prior therapies. The ORR was 46% (3 CR and 13 PR), with an overall median duration of response of 4.91 months (range 0.9–26.6). In the three PTCL patients who achieved CR, the median response duration was 19.5 months [59]. A phase I combination trial with romidepsin is ongoing (NCT03770000). While the P13K inhibitors have struggled to find a consistent role in the B-cell disorders, the combination therapies in T-cell lymphomas are so far promising and merit further scrutiny in larger cohorts.

### 3.3. JAK/STAT and Syk Pathway Inhibitors

The Janus kinases (JAK1-3) allow the phosphorylation of cytokine receptors via their tyrosine kinase activity, resulting in the activation of the downstream STAT (signal transducer and activator of transcription) family members, leading to cellular proliferation. Aberrancies in the JAK/STAT pathway are common in T-cell lymphomas, with one series reporting an association with worse outcomes in PTCL [60]. The JAK/STAT pathway is also a potential mediator of pruritus in CTCL [61]. The tyrosine kinase Syk is a driver of cellular growth and survival in healthy and malignant B-cells and not usually expressed on normal T-cells. However, it has been shown to be aberrantly expressed in most PTCLs, with in vitro silencing leading to apoptosis of PTCL cells [62].

A phase II trial of the JAK2 inhibitor ruxolitinib in R/R PTCL and CTCL reported an ORR of 23%, with responses more frequent in the AITL and Nodal Peripheral T-Cell Lymphoma with TFH Phenotype PTCL-TFH) subtypes [63]. Cerdulatinib is a small-molecule reversible ATP competitive inhibitor of SYK and JAK family members. It was shown to demonstrate activity in a cohort of R/R PTCL (ORR 35%) and CTCL (50%), with reportedly rapid improvement in pruritus [64].

### 3.4. Interleukin-2-Inducible T-Cell Kinase (ITK)

The TEC family tyrosine kinases mediate intracellular signaling in hematopoietic cells. They include TEC (tyrosine kinase expressed in hepatocellular carcinoma), BTK (Bruton’s tyrosine kinase), ITK (IL2-inducible T-cell kinase), RLK (Resting lymphocyte kinase), and BMX (bone marrow-expressed kinase). ITK is a T-cell specific tyrosine kinase essential for signaling from the T-cell receptor (TCR) and chemokine-induced migration. ITK deleted mice show defects in CD4+ T-cell differentiation [65,66]. The BTK inhibitor ibrutinib has off-target ITK inhibition but no significant activity in T-cell lymphoma [67]. CPI-818 is a first-in-class irreversible ITK inhibitor with selectivity for ITK. A phase I dose-escalation trial with biomarker assessment is ongoing and recently reported on 16 patients with PTCL or CTCL. The drug was well tolerated and achieved almost complete ITK inhibition at target dose. One of the four PTCL patients obtained a CR and clinical benefit seen in three of the seven CTCL patients, including a nodal CR [68].

## 4. The Epigenome: Histone Deacetylase Inhibitors and Hypomethylating Agents

Epigenetic changes in tumors often result in inappropriate gene silencing and effect a diverse variety of cellular functions affecting the microenvironment and immune response. They are the result of clonal changes in gene expression that are mediated by mechanisms that do not alter the DNA sequence. Epigenetic alterations are found not infrequently in T-cell lymphomas and are generally either the result of loss or gain of function mutations in genes regulating histone modification or DNA methylation. These mutations in the T-cell lymphomas epigenome are the prime target of the histone deacetylase inhibitors and the hypomethylating agents; however, other agents active in T-cell lymphoma, such as the folate analogue pralatrexate, have demonstrated via gene expression profiling (GEP) an ability to modulate the expression of genes involved in DNA methylation and apoptosis [69].

### 4.1. Histone Deacetylase Inhibitors

Histone deacetylase inhibitors (HDACi) interact with a range of targets from the intrinsic molecular processes of the tumor to its surrounding microenvironment, resulting in potent anticancer activity. They exert their influence via both modification of histone mediated transcription and non-histone effects such as modification of transcription factors and other regulatory proteins. HDAC inhibitors are classified according to their chemical structure, and each varies in its ability to inhibit individual HDACs [70].

Vorinostat, a pan-HDAC inhibitor, was the first HDACi approved by the FDA for the treatment of CTCL. A phase II trial of vorinostat 400 mg daily showed a partial response in 22 (29.7%) of 74 patients with R/R CTCL, with only one CR. Another phase II trial assessed several dosing regimens with 300 mg twice daily reported to have greater levels of toxicity without superior efficacy when given to 33 R/R CTCL vorinostat produced an ORR of 24.2% (eight patients), with almost half of patients reporting significant improvement in quality of life due to reduction in pruritus. However, when compared to mogamulizumab in the MAVORIC trial, as described previously, the ORR was low, at 4.8% [38,71].

The first HDACi approved for use in PTCL was the bicyclic class 1 selective HDACi romidepsin, which was based on a phase II study of romidepsin monotherapy in 130 patients with PTCL. The reported ORR was 25% with a median PFS of 29 months. The most common AEs were gastrointestinal and hematological disturbances with cardiac arrhythmias uncommon [72].

Perhaps unsurprising, the PTCL subtypes characterized by the T follicular phenotype and defined by hallmark mutations in epigenetic regulators, namely AITL and PTCL-TFH, have been shown to more responsive to HDAC inhibition. A retrospective cohort of 164 patients reported higher response rates in patients with TFH phenotype (ORR 56.5%) than those with and in non-TFH phenotype patients (ORR 56.5% vs. 29.4% *p* = 0.0035) [73].

Romidepsin is also approved for R/R CTCL supported by two phase II prospective trials that included 71 and 96 patients, respectively, with both studies reporting an ORR of around 35% [74,75]. The second HDACi to receive FDA approval for the treatment of R/R PTCL was the pan-HDAC inhibitor Belinostat. The response rates reported in the phase II ‘BELIEF’ study were similar to those of romidepsin with an ORR of 26% and a median duration of response of 13.6 months. The toxicity profile appears to be slightly more favorable than romidepsin with the most common grade 3/4 adverse events being anemia (10.8%) and thrombocytopenia (7%).

Despite encouraging single-agent activity, the addition of romidepsin to conventional chemotherapy was disappointing, with a phase III trial of romidepsin and CHOP being unable to demonstrate superiority when compared to CHOP with regards to PFS and OS in R/R PTCL patients [76]. Combination therapy with novel agents, however, is proving more promising, with the combination of romidepsin with pralatrexate proving to be potent in a phase I trial of R/R PTCL. Eighteen patients with PTCL with a median number of three prior systemic treatments were included. The ORR was 71%, with four patients (29%) obtaining a CR. The most common grade 3 adverse effects were anemia (29%) and oral mucositis (14%) [77]. Discussed elsewhere are other combinations potentially exhibiting synergistic efficacy with romidepsin, including azacitidine, lenalidomide, and the P13K inhibitors.

### 4.2. Hypomethylating Agents/DNA Methyltransferase Inhibitors

The hypomethylating agents or DNA methyltransferase inhibitors (DNMTi) azacitidine and decitabine are pyrimidine nucleoside analogues of cytosine and can become incorporated into DNA forming covalent bonds between the 5-azacytosine ring and the DNMT enzyme, thus causing irreversible DNMT inactivation. It is thought they likely reverse the hypermethylation that results in silencing of tumor-suppressor genes.

Lemonnier et al. reported on 19 patients with R/R PTCL treated with azacitidine with an enrichment of patients with the AITL subtype (12 patients). The ORR was 53% in the cohort but was 75%, with a CR rate of 50% in the AITL patients, which characteristically have mutations in epigenetic drivers such as TET2 [78]. A recent phase II study combined oral azacitidine with romidepsin in 25 patients with R/R PTCL and reported an ORR of 61% with an impressive CR of 48% in a heavily pretreated population. Favorable response was associated with the presence of mutations in DNA methylation and histone deacetylation genes. [79]. The combination of azacitidine with conventional chemotherapy is also being examined, with an ASH 2020 update poster reporting interim results demonstrating the feasibility of an azacitidine and CHOP chemotherapy combination. Enrolment prioritized PTCL subtypes defined by epigenetic molecular aberrancies, such as AITL, and the study reported an impressive CR rate of 76.5% at the end of treatment [80]. This combination is now planned for comparison in duvelisib–CHOP in a randomized trial in CD30 negative PTCL. As certain subtypes of T-cell lymphomas are increasingly defined by the presence of epigenetic mutations, it is clear that the role for the hypomethylating agents is expansive.

## 5. Immunotherapy and Immunomodulators

### 5.1. Checkpoint Inhibitors

Programmed death ligand 1 (PD-L1) is overexpressed in the microenvironment of the T-cell lymphomas and is a rational therapeutic target although the initiation of tumor hyperprogression is a potential concern. The PD1 and PDL-1 inhibitors, rapidly expanding as a class of drugs due to their efficacy in solid organ cancers, have been trialed in a range of hematological malignancies. Modest single-agent efficacy has been demonstrated so far, except for the NK-T cell lymphomas, which appear markedly sensitive to PD1 inhibition [81,82] (see below). The adverse-effect profile reported seems to be in keeping with treatment of solid organ tumors where their use is more widespread; however, there have been reports of hyperprogression [83].

A phase I trial of the PD-1 inhibitor nivolumab in patients with hematological malignancy included 13 patients with mycosis fungoides (MF), five with PTCL, and five with ‘other’ T-cell lymphomas. The ORR was 17%, with no patients obtaining a CR. The subsequent phase II trial was halted early due to concerns with hyperprogressive disease [83,84]. A phase II trial of the PD-1 inhibitor pembrolizumab was able to demonstrate somewhat better results in eighteen patients with a median line of two prior treatments. The ORR was 33% with four patients (27%) responding obtaining a CR. Two of the four responses were still ongoing at >9 months, with two patients coming off the study for toxicity and transplantation [81]. Combination therapies are underway, and early results are perhaps more encouraging. A phase I/II trial combining pembrolizumab with romidepsin in 15 patients with R/R PTCL demonstrated an ORR of 44%, with three patients obtaining a CR, all of whom remain in remission after 10 months [85]. Other combinations currently in trial are PD-1 or PD-L1 inhibitors in combination with pralatrexate, decitabine, azacitidine, and lenalidomide.

While the utility of PD1/PD-L1 inhibition in most T-cell lymphomas appears underwhelming so far, the PD-1 inhibitors have been demonstrated to be highly effective at salvaging relapsed or refractory NK/T-cell lymphoma. A rare and aggressive PTCL subtype associated with Epstein Barr virus and endemic in East Asia, salvage therapies are typically ineffective and progression after frontline L-asparaginase therapy is generally fatal [86]. Kwong et al. reported seven patients that had all relapsed post frontline SMILE (dexamethasone, methotrexate, ifosfamide, l-asparaginase, and etoposide) or SMILE-like treatment. The ORR was 100%, with five of the seven patients obtaining a durable CR [82]. Further retrospective studies have supported this reported efficacy and prospective trials are underway [87]. Recent data published have demonstrated via whole-genome sequencing that structural alterations of PD-L1 are seen in patients with NK/T-cell lymphoma who respond to pembrolizumab [88].

### 5.2. Immunomodulators

The Immunomodulatory drugs or ‘IMiDS’ have been a cornerstone of multiple myeloma therapy since the FDA approval of thalidomide for newly diagnosed myeloma in 1998. Exerting their effect on immune cells primarily by inhibition of the *E3 ubiquitin ligase* cereblon, thalidomide and its derivatives, namely lenalidomide and pomalidomide, have a widespread effect on the immune system and display activity in numerous hematological malignancies [89].

The phase II EXPECT trial demonstrated the single-agent activity of lenalidomide in a heavily pretreated cohort of 54 patients with R/R PTCL, including 26 with AITL, where lenalidomide seemed to be more active. The overall ORR was 22% (31% in AITL), with 11% of patients obtaining a CR. The ORR in AITL was 31% [90].

IMiD-chemotherapy combination trials are planned or ongoing. The combination of lenalidomide with romidepsin was able to demonstrate significant activity in PTCL and CTCL with ORRs of 50% and 56%, respectively, in 21 patients with T-cell lymphoma (11 PTCL 10 CTCL). The combination of lenalidomide with vorinostat has been also trialed and reported more modest responses with an ORR of 25% in eight patients. [91,92]. In the rare prospective upfront setting, a recent trial of 153 patients was reported demonstrating superiority of GDPT (gemcitabine, cisplatin, prednisone, and thalidomide) versus CHOP in previously untreated patients with PTCL, with respect to ORR (66.3% vs. 50.0%, *p* = 0.042), CR rate (42.9% vs. 27.6%, *p* = 0.049), 4-year PFS (63.6% vs. 53.0%, *p* = 0.035), and 4-year OS (66.8% vs. 53.6% for OS, *p* = 0.039). There were no differences in measured adverse effects between the two cohorts, with myelosuppression being the most common grade 3 or 4 AE [93].

### 5.3. Chimeric Antigen Receptor T-Cell Therapy

Chimeric antigen receptor T-cells (CAR-T) are among the most promising novel therapies with remarkable responses demonstrated in patients with B-cell malignancies in even the most refractory or relapsed settings. CAR T-cell constructs are created by inserting a receptor with a specific antigen-binding domain into an autologous T-cell, using a viral vector. This binding domain is expressed as a high-affinity tumor-specific antigen receptor on the surface of the CAR T-cells, allowing the CAR T-cells to exhibit direct antitumor activity, which is enhanced as they undergo in vivo expansion. Despite the promising results in B-cell malignancies, CAR T-cell therapy in the setting of T-cell malignancies faces multiple additional hurdles, not the least being the selection of a T-cell target that averts fratricide of the CAR T-cells themselves [94,95].

Multiple preclinical studies have evaluated different antigens as the target for CART including CD3, CD4, CD5, CD7, and CD30. The most promising target so far is CD30, with Ramos et al. reporting a cohort of patients with CD30+ lymphoma, predominately Hodgkin’s lymphoma, that included a patient with ALK+ ALCL that achieved a CR that persisted for nine months [96,97]. Other groups are examining CAR T-cell antigens in clinical trials that may include T-cell lymphomas such as anti-CD5 CART and the CAR NK cell anti-CD7 CAR-pNK.

### 5.4. Bispecific Antibodies

The bispecific T-cell engager (BiTE) antibodies are fusion proteins consisting of two single-chain variable fragments of different antibodies, one specific to a tumor-cell antigen and the other a T-cell receptor, usually CD3, which promotes T-cell cytotoxic activity against the tumor cell independent from MHC and co-stimulatory pathways. For similar reasons as to CAR-T therapy, it has been more difficult to design similar antibodies against T-cell lymphoma cells.

AFM13 is an investigational CD30/CD16A bispecific antibody that engages and activates NK cells and macrophages. It has demonstrated activity in R/R Classical Hodgkin’s lymphoma with a tolerable safety profile [98]. A phase Ib/IIa trial in patients with R/R CD30+ T-cell lymphoma with cutaneous presentation in 15 patients, including 5 with a PTCL variant, reported a poster at ASH 2020. The ORR was 42% with responders including those with prior brentuximab exposure. Toxicity was mostly grade 1 infusion reactions with grade 3 skin infections seen in two patients. In R/R Classical Hodgkin’s lymphoma AFM13 has been shown to demonstrate modest activity (ORR 23%), but when combined with PD1 inhibition, response rates were much improved (ORR 83%) [99], perhaps beyond what is expected of PD1 inhibition alone and suggestive of synergism; however, to date, no trials have been reported in CD30+ T-cell lymphoma.

## 6. Conclusions

The future of T-cell lymphoma management was previously bleak, with management restricted to chemotherapy regimens largely adopted from their B-cell counterparts. Despite attempts to improve the durability of responses with the addition of further chemotherapy agents and stem-cell transplantation, the outcomes were unsatisfactory for many patients. There is increasing recognition that the T-cell lymphoma subtypes are perhaps even more different than they appear, with vast molecular and genetic heterogeneity between them (Table 2). This is reflected in the divergent responses seen between the subtypes to different targeted therapies. The refinement of the molecular drivers of lymphogenesis in different subtypes and the subsequent therapeutic benefit of inhibition have begun the divergence from the B-cell lymphomas. The novel targeted therapies, in combination with either each other or conventional chemotherapy, have been able to demonstrate remarkable responses in even the most relapsed or refractory cases and signaled that, in the future, therapeutic choices may be tailored to the attributes of both the lymphoma and the patient—true ‘personalized medicine’.

## Figures and Tables

**Table 1:** Prospective trials of CD30 antibody–drug conjugate Brentuximab Vedotin in T-cell lymphoma.

Trial	Intervention	No. of Patients	Subtype	Patient Population	Study Design	ORR%	CR%	PFS	OS
Horwitz et al. [7]	BV	35	13 AITL; 22 PTCL-NOS	Relapsed/Refractory	Phase II	41	23.5	Median PFS 2.6 months	NR
Pro et al. [8]	BV	58	ALCL ALK + 28%; ALK − 72%	Relapsed/Refractory	Phase II	86	57	5-year: 39%	5-year: 60%
Horwitz et al. [9]	BV + CHP vs. CHOP	226 vs. 226	BV-CHP: sALCL 72%; non-ALCL 28% CHOP: sALCL 68%; non-ALCL 22%	Frontline	Phase III	83 vs. 72	68 vs. 56	3-year: 57 vs. 44%	Median OS not reached
Kim et al. [10]	BV	32	CTCL 29 MF; 3 SS	Relapsed/Refractory	Phase II	70	3	12 months: 79%	NR
Duvic et al. [11]	BV	48	CTCL CD30 + LPD 28; MF/SS 20	Relapsed/Refractory	Phase II	73CD30 + LPD 100; MF/SS 54	35	Median PFS: 1.1 years	Median OS not reached
Prince et al. [12,13]	BV vs. physician’s choice (PC)	64 vs. 64	CTCL BV: MF 75%; pcALCL 25% PC: MF 77%; pcALCL 23%	Relapsed/Refractory	Phase III	56.3 vs. 12.5 (ORR4)	16 vs. 2	Median PFS: 16.7 vs. 3.5 months	NR

BV, Brentuximab Vedotin; NR, not reported; AITL, angioimmunoblastic T-cell lymphoma; PTCLNOS, PTCL not otherwise specified; ALCL, anaplastic large-cell lymphoma; sALCL, systemic ALCL; pcALCL, primary cutaneous ALCL; CD30+ LPD, CD30+ lymphoproliferative disorder, MF, mycosis fungoides; SS, Sezary syndrome; ORR4, objective response lasting at least four months.

**Table 2:** **Targeted T-cell therapy by T-cell lymphoma subtype.** +++ Data available suggesting particular benefit; + data support routine use; +/− data indeterminate or further data required; − no data to support routine use.

	PTCL-NOS	AITL	sALCL ALK+	sALCL ALK-	MF	SS
Brentuximab Vedotin	+	+	+++	+++	+	+
Alemtuzumab	+/−	+/−	+/−	+/−	+/−	+
Mogamulizumab	+/−	+/−	+/−	+/−	+/−	+
Denileukin diftitox	+/−	+/−	+/−	+/−	+	+
ALK inhibitors	−	−	+++	−	−	−
PI3K inhibitors	+/−	+/−	+/−	+/−	+/−	+/−
JAK/STAT inhibitors	+/−	+/−	+/−	+/−	+/−	+/−
HDAC inhibitors	+	+++	+	+	+	+
Hypomethylating agents	+	+	+	+	+/−	+/−
